# Quality of Life Assessments in Individuals With Young-Onset Dementia and Their Caregivers

**DOI:** 10.1177/0891988720933348

**Published:** 2020-07-09

**Authors:** Divyansh Dixit, John Spreadbury, Rosanna Orlando, Elaine Hayward, Christopher Kipps

**Affiliations:** 1 12211 Faculty of Medicine, University of Southampton, United Kingdom; 2 130346 NIHR Applied Research Collaboration Wessex, Southampton, United Kingdom; 3 Unisalento, Department of Economics, Lecce, Italy; 4 Wessex Neurological Centre, 7423 University Hospital Southampton, Southampton, United Kingdom

**Keywords:** dementia, quality of life, young-onset dementia, caregiver burden, caregiver

## Abstract

**Background:**

Quality of life (QoL) has seldom been investigated or explicitly measured in young-onset dementia (YoD). The aims of this study were (1) to investigate and compare QoL self- and proxy reports in a sample of YoD patients and caregivers using different conceptual assessments of QoL and (2) to examine the relationship between caregiver QoL and both burden and mental health.

**Methods:**

There were 52 participants (26 YoD patient–caregiver dyads). The design was cross-sectional and part of a larger longitudinal prospective cohort study of YoD patients and caregivers. Primary measures included generic QoL (World Health Organization Quality of Life–short version [WHOQOL-BREF]), dementia-specific QoL (Quality of Life in Alzheimer’s Disease Scale [QoL-AD]), health-related QoL (EQ5D), and a single-item QoL measure. Secondary measures included caregiver burden (Zarit Burden Index), mental health (Hospital Anxiety and Depression Scale), and dementia severity (Clinical Dementia Rating).

**Results:**

Patient QoL self-reports were higher than caregiver proxy reports on the QoL-AD (*P* = .001). Patient QoL self-reports for the WHOQOL-BREF (*P* < .01) and single-item QoL (*P* < .05) measure were significantly higher than caregiver self-reports. Dementia severity had no relationship with QoL self-reports. Caregiver burden, anxiety, and depression were negatively correlated with QoL when measured using a generic and single-item measure, but not with the health-related measure.

**Discussion:**

Patients and caregivers show a disparity in QoL reports, with patients tending to report higher QoL. Caregiver burden, anxiety, and depression should be areas targeted for interventions when supporting caregivers.

## Introduction

Young-onset dementia (YoD), also known as early-onset dementia, refers to the emergence of symptoms of dementia under the age of 65 years. Young-onset dementia may differ from late-onset dementia (LoD) in several ways and typically presents with a wider range of symptoms than memory disturbance, including other domains such as behavior, language, and perceptual disturbance.^1-3^ Alzheimer's disease (AD) is considered the most prevalent dementia subtype in both YoD and LoD, although represents a smaller proportion of young-onset cases (YoD: 34%; LoD: 62%^4,5^).^2-6^ In contrast, other dementia subtypes such as frontotemporal dementia (FTD) are seen more frequently^1-5^ (YoD: 12%; LoD: 2%^4,5^).

Dementia in earlier life may cause significant psychosocial problems for patients and their families and may negatively impact areas of life such as employment, parenting, relationships, finances, retirement planning, future aspirations, and sense of self.^7^ Furthermore, there may be additional problems around stigma and reduced contact with friends.^8-10^ Mid-life dementia places particular stress on family life. There may be conflicts^11^; children with a YoD parent may struggle to understand and cope with their experience,^12^ while primary carers, often spouses or partners, can be at particular risk of caregiver burden and mental health problems.^13^ Young-onset dementia can also be challenging for health and social care professionals, particularly around the areas of assessment, diagnosis, and provision of suitable community care, particularly where these are predominantly offered to an older age-group.^14^ Although there has been advancement in understanding the subjective experience of patients and carers in YoD,^15,16^ until recently there had been scant explicit measure of their quality of life (QoL).^15-17^ Recent reviews suggests the majority of dementia care is focused on patients with LoD and have recommended QoL as an important area of psychosocial experience to investigate in younger populations.^15-17^

### Quality of Life

Different theoretical frameworks have been used to define, conceptualize, and measure QoL. A “generic” conceptualization takes a holistic perspective and focuses on measuring multiple dimensions of a person’s life such as their physical and psychological well-being, relationships with other people, and satisfaction within the environment in which they live.^18,19^ This approach may be considered a generic measure as it could be applied across cohorts and contexts. In contrast, a “specific” conceptualization may be more unidimensional and focused on measuring only one aspect of a person’s life such as their health and is best represented by health-related QoL measures.^18^ There may also be a “disease-specific” conceptualization that focuses on measuring QoL in the context of a specific health condition such as cancer or dementia.^18^

“An authoritative and influential generic definition of QoL comes from the World Health Organization Quality of Life Group and defines QoL as follows: “individuals' perception of their position in life in the context of the culture and value systems in which they live and in relation to their goals, expectations, standards, and concerns.” It is a broad ranging concept affected in a complex way by the persons’ physical health, psychological state, level of independence, social relationships, and their relationship to salient features of their environment”.^19^ Regardless of the conceptualization or measure employed, the task of participants is usually to subjectively indicate the level of “goodness”^20^ of their life, different areas of their life, or in the case of proxy ratings, somebody else’s life.

### Quality of Life in YoD

Recent reviews indicate that up until recently there had been very little research specifically addressing and measuring QoL in a younger population with dementia.^15,16^ However, the last 3 or 4 years has seen QoL become the focus of significant research efforts in YoD. Research from 2 large European prospective cohort studies using caregiver proxy reports of patient QoL, measured using the Quality of Life in Alzheimer’s Disease Scale (QoL-AD),^21^ identified variables such as depression, met and unmet needs, and impaired patient awareness that can have a negative influence on QoL,^22-24^ as well as suggesting a lack of difference in QoL between YoD subtypes (e.g. AD compared with FTD).^22-24^ In addition, earlier research using the QoL-AD and RAND-36 identified that depression negatively influenced health-related QoL in patients. In caregivers, unmet needs of both patients and caregivers negatively influenced caregiver health-related QoL.^25^ Other findings including group comparisons, reveal that patients can perceive their QoL to be better than caregiver proxy reports,^25,26^ while YoD caregivers score lower on QoL than those providing care to individuals with LoD.^27^

Despite progress, a lack of empirical studies remains and a number of methodological drawbacks are evident. There appears to be a tendency to use caregiver proxy reports with a scarcity of self-reports by patients. Indeed, more generally, there is some uncertainty about the level of patient involvement in YoD research.^15^ In addition, there are few comparisons between patient and caregiver self-reports and little use of QoL instruments beyond the QoL-AD—a measure widely used in LoD^18^ but not necessarily suitable in capturing the QoL challenges pertinent to YoD. However, at present and to the best of our knowledge, there is no QoL measure dedicated to YoD.

## Aims

The aims of the present study were to investigate QoL in YoD patients and caregivers, to compare patient and caregiver self- and proxy reports utilizing the principal conceptual assessments of QoL (i.e. broad/generic, disease-specific, health-related, and a single-item metric), and to explore the clinical utility of different QoL measures in a YoD cohort. An additional aim was to investigate the relationship between caregiver QoL and 2 of the most significant risk factors for caregivers, namely caregiver burden and mental health problems (anxiety and depression).

## Method

### Participants

There were 52 participants (26 YoD patient–caregiver dyads). Age of symptomatic onset and diagnosis for all patients was before the age of 65 years. Dementia subtypes were as follows: 17 (65%) AD, 5 (19%) FTD, and the remaining 5 (19%) had other dementia subtypes, for example, primary progressive aphasia. The mean age of patients was 59.0 years (SD: 4.7), with 15 males and 11 females, all of whom were community dwelling and supported by caregivers. Of the 26 carers, 23 were spouse/partners living with the patient, 2 were family members, neither of whom lived with the patient (daughter and brother), and 1 was a close friend living with the patient. Participants were recruited from an outpatient clinic for cognitive disorders in a large hospital in the South of England.

### Design

This was a cross-sectional study and part of a broader longitudinal prospective cohort study of YoD patients and caregivers. Research Ethics Committee ethical approval was obtained and all participants had mental capacity to provide written informed consent which was obtained in all cases.

### Materials

All measurement instruments utilized are widely used with satisfactory validity and reliability. Multiple measures of QoL were employed, including dementia-specific, generic, health-related, and a single-item metric. Additional measures included caregiver burden and mental health.

#### Dementia-specific QoL

Dementia-specific QoL was measured using the QOL-AD.^21^ The scale assesses patient self-reports and caregiver proxy reports of QoL specific to dementia. It has 13 items with each one addressing a separate area of QoL (e.g. physical, energy, mood, living, memory, family, marriage, friends, self-assurance, chores, fun, money, and life). The scale has good psychometric properties, appropriate for varying disease stages,^28^ and uses a 1 (poor) to 4 (excellent) response format producing one overall QoL score between 13 and 52.

#### Generic QoL

Generic QoL was measured using the World Health Organization Quality of Life–short version (WHOQOL-BREF).^29^ This is a holistic assessment and measures QoL in 4 specific domains: physical health, psychological health, social relationships, and environment.^30^ The scale has 26 items and uses a 1 to 5 response format. Scores can be formulated to produce an overall score and for each of the individual domains. The scale is derived from the WHOQOL 100-item questionnaire, has good psychometric properties,^31^ is widely validated,^32^ and has been used cross-culturally.^30^

#### Single-item QoL

A single-item QoL measure was used to complement the multi-item QoL measures used and to investigate the utility of measuring QoL with a single item.^20,33^ The item is a simple QoL self-report measure used successfully in gerontological research^33^ and clinical practice,^34^ consisting of the solitary question: “Thinking about the good and bad things that make up your QoL, which of the answers below best describes the quality of your life as a whole?” The response format for this item used a 7-point Likert scale ranging from 1 to 7,^20^ offering a quick and simple self-report of QoL, measuring QoL without emphasis on particular subdomains.^20^

#### Health-related QoL

Health-related QoL was measured using the health status/health state instrument, the EQ5D.^35,36^ This scale consists of 5 items assessing mobility, self-care, activities, pain/discomfort, and anxiety/depression. The EQ5D is considered a valid and reliable measure of health-related QoL^37^ and has been used with people with dementia and their caregivers.^38,39^ Scores are converted to form an overall Visual Analog Score (VAS) 0 to 1.^40^

#### Caregiver burden

Caregiver burden was measured using the Zarit Burden Interview (ZBI).^41^ This scale is a frequently used measure of burden in caregivers.^42^ It has validity and reliability in measuring caregiver burden in people with disability,^43^ especially dementia.^42^ It is appropriate for a variety of populations, as it is unrelated to age, gender, marital status, and living and employment status.^42^ It consists of 22 items, each with a 5-point response format 0 to 4, which are summed to provide an overall score of 0 to 88. Scores can be grouped to form the following burden level categories: 0 to 20 (none/little), 21 to 40 (mild/moderate), 41 to 60 (moderate/severe), and 61 to 88 (severe).

#### Mental health

Caregiver mental health was measured using the Hospital Anxiety and Depression Scale (HADS).^44^ This scale is a frequently used research and clinical tool used in detecting the presence and severity of anxiety and depression symptomology. It consists of 14 items, half assessing anxiety and half depression, each with a 4-point response format (0-3). Scores for each item are totaled and the following indicative diagnostic categories can be used: 0 to 7 (normal), 8 to 10 (borderline case), and 11 to 21 (case). The scale is shown to be a valid measure in monitoring symptom progression^45^ among a wide range of patients within the general population^46^ and is a National Institute for Health and Care Excellence recommended diagnostic tool.^45^

#### Dementia severity

Dementia severity was measured using the Clinical Dementia Rating (CDR). It offers a measure of stage/severity of cognitive impairment, in this case completed by the caregiver.^47^ It is shown to be a valid method to determine disease stage compared to a gold standard.^48^ There are 6 items corresponding to memory, orientation, judgment/problem-solving, community affairs, home/hobbies, and personal care. Each question has a 5-point scale, and the final CDR rating is calculated: normal (0), very mild (0.5), mild (1), moderate (2), and severe (3).^49^

All questionnaires were completed as follows (see Table 1): Patients self-reported their own QoL for the QoL-AD, WHOQOL-BREF, single-item QoL, and EQ-5D. Caregivers provided proxy reports evaluating patient QoL using the QoL-AD and dementia severity using the CDR. Caregivers self-reported their own QoL using the WHOQOL-BREF, single-item QoL, and EQ-5D. Caregivers self-reported their own caregiver burden using the ZBI and mental health using the HADS. For all QoL measures, higher scores indicated higher (or better) QoL. For the ZBI and HADS, higher scores indicated higher caregiver burden and depression/anxiety symptomatology, respectively.

**Table 1.** List of Questionnaires and Respondents.

Questionnaire	Patient self-report	Caregiver self-report	Caregiver proxy report about patient
QoL-AD	✓		✓
WHOQOL-BREF	✓	✓	
Single-item QoL	✓	✓	
EQ-5D	✓	✓	
HADS		✓	
Zarit Burden Interview		✓	

Abbreviations: HADS, Hospital Anxiety and Depression Scale; QoL, quality of life; QoL-AD, Quality of Life in Alzheimer’s Disease Scale; WHOQOL-BREF, World Health Organization Quality of Life–short version.

### Procedure

The data were collected by a research nurse visiting patients and caregivers in their own homes. Participant responses were recorded on paper copies of scales and then transferred into an electronic platform for management and storage. Double data entry was used to ensure data accuracy, and data were exported into SPSS Ver25.0 (IBM Corp) for statistical analysis.

### Analysis

A high proportion of questionnaires were completed: QoL-AD (96.2%), WHOQOL-BREF (94.2%), single-item QoL (100%), EQ5D (100%), ZBI (92.3%), and HADS (96.2%). Mean imputation was used to manage missing data. Although research suggests using mean imputation may distort relationships between variables,^50^ this was deemed a minor concern and the most suitable method due to the scarcity of missing data throughout the sample.

Normality assumptions were tested using Kolmogorov-Smirnov tests, confirmed visually via histogram plots. Questionnaire data were parametric except for the single-item QoL and EQ5D. Patient WHOQOL-BREF data showed a borderline nonparametric/parametric distribution (Kolmogorov-Smirnov = 0.051), but visual assessment supported a parametric distribution.

Descriptive statistics are reported in the format: mean (SD) for parametric data and median (range) for nonparametric. Descriptive statistics are presented for patient/caregiver groups for all questionnaires, except the ZBI and the Depression/Anxiety parts of the HADS, which are exclusively completed by caregivers (Table 1).

The parametric data sets for patient QoL-AD self-reports and caregiver QoL-AD proxy reports are compared using a paired-sample *t* test as were patient and caregiver self-reports for the WHOQOL-BREF. Nonparametric Wilcoxon signed-rank tests were used to compare patient and caregiver self-reports for the single-item QoL and EQ5D dyad data sets.

Analyses of 3 carer self-measures of QoL against 3 QoL-factors (caregiver burden, anxiety, and depression) were performed. Both ZBI and HADS scores were correlated with each using Spearman rank correlation. Outcome groups from the HADS were derived (case, borderline, normal), which were used in the analysis of QoL via Kruskal-Wallis test. Post hoc *t* tests or Mann-Whitney *U* tests identified group differences where relevant.

## Results

### Dementia-Specific QoL (QoL-AD): Patient Self Report Versus Caregiver Proxy Report

Patients self-reported their own QoL as being significantly higher than caregiver proxy reports using the QoL-AD: mean 35.7 (SD: 6.9) versus 29.0 (SD: 6.4); *t*(48) = 3.549, *P* = .001, see Table 2.

**Table 2.** Patient and Carer scores on QoL Measures including WHOQOL Subdomains.

Domain	Patient		Carer		Independent *t* test	
	Mean	SD	Mean	SD	*t*	Significance
QOL-AD	35.7	6.9	29.0	6.4	3.55	.001
WHOQOL-BREF total	102.6	13.2	94.1	15.8	2.02	.049
WHOQOL-BREF physical	73.4	15.8	67.4	21.3	1.10	.278
WHOQOL-BREF psychological	69.9	14.0	59.2	14.0	2.54	.015
WHOQOL-BREF social	74.7	19.3	59.4	20.2	2.68	.010
WHOQOL-BREF environmental	79.0	12.8	72.0	16.1	1.66	.104
	Median	Range	Median	Range	Mann-Whitney test (significance)	
Single-item QOL	5.0	2.0-7.0	4.0	3.0-6.0	.028	
EQ5D	0.7	0.26-1.0	0.7	0.17-1.0	.680	

Abbreviations: QoL, quality of life; WHOQOL-BREF, World Health Organization Quality of Life–short version.

### Generic, Single-Item, and Health-Related QoL: Patient Self-Report Versus Caregiver Self-Report

The mean self-report score for patients was significantly higher compared with caregiver self-reports using the WHOQOL-BREF: mean 102.6 (SD: 13.2) versus 94.1 (SD: 15.8); *t*(23) = 2.02, *P* < .05; see Table 2. Subdomain analysis (physical, psychological, social, and environmental) identified significantly higher (better) scores in psychological and social domains for patients compared with caregivers (see Table 2).

On the single-item measure of QoL, patients reported their QoL as being significantly higher than caregiver self-reports: patient median score 5.0 (range = 2-7) corresponding to a rating of “good,” versus caregiver median score 4.0 (range = 3-6) corresponding to a rating of “alright” (*Z* = −2.2, *P* = .028), see Table 2. On the health-related QoL measure (EQ5D), there was no significant difference between patient and caregiver self-reports: patient median 0.7 (range = 0.26-1.00), caregiver median 0.69 (0.17-1.00), *P* = .68.

### Caregiver QoL, Burden, and Mental Health

The mean caregiver burden score on the ZBI was 35.1 (SD: 18.2), indicating mild/moderate burden. Caregiver responses were split into 3 groups: “none/little” burden, “mild/moderate” burden, and “moderate/severe” burden (see Table 3 for details of each group). Two caregivers did not complete the ZBI. Total burden scores were negatively correlated with measures of self-rated QoL: WHOQOL-BREF (Spearman rank correlation = −0.619, *P* < .01) and single-item QoL (−0.584, *P* < .01), but not health-related QoL using the EQ5D (−0.145, *P* > .50). Mean testing using QoL as the dependent variable comparing caregiver (ZBI) burden groups showed a significant difference for the single-item QoL measure (Kruskal-Wallis *H*(2) = 6.332, *P* < .05), but not for the WHOQOL-BREF (1-way analysis of variance *F*_2_ = 2.67, *P* > .05). Post hoc Mann-Whitney *U* tests showed that for the single-item QoL measure, those with none/little burden (mean single-item QoL = 5.6) had significantly higher mean QoL scores compared with moderate/severe or severe burden (3.9; Z = −2.20, *P* < .05).

**Table 3.** Group Data: Zarit Burden Interview (ZBI) and Hospital Anxiety and Depression Score (HADS).

	Questionnaire classification	n	%	Mean
ZBI caregiver burden	None/Little	6	25	14.8
	Mild/Moderate	10	42	33.0
	Moderate/Severe	8	33	58.3
HADS caregiver depression	Normal	18	72	4.7
	Borderline case	4	16	8.5
	Case	3	12	13.0
HADS caregiver anxiety	Normal	12	48	3.7
	Borderline case	7	28	9.6
	Case	6	24	14.0

We conducted 2 hierarchical multiple regression models using WHOQOL-BREF as the dependent variable in the first and single-item QoL in the second. Caregiver burden was used as an independent predictor, controlling for dementia severity. In both models, dementia severity was non-significant but accounted for a small percentage of the variance in QoL (WHOQOL-BREF: 9%; single-item QoL: 12%). Caregiver burden, entered as an independent predictor, was significant (WHOQOL-BREF: *F*_1,16_ = 9.76, *P* = .007; single-item QoL: *F*_1,15_ = 9.24, *P* = 0.008), accounting for 34.4% and 33.5%, respectively.

Using the HADS to assess depression and anxiety in caregivers, each was classified as “normal,” “borderline case,” and “case” (13.0), see Table 3 for details. One caregiver did not complete the HADS questionnaire. Correlation of anxiety and depression total scores, on the HADS, with carer QoL self-reports found a significant relationship with WHOQOL-BREF and single-item QoL, but not the EQ5D (see Table 4). We conducted means testing comparing QoL (dependent) against depression and anxiety (grouping variables). Means comparison tests showed depression groups scored similarly for all QoL measures (WHOQOL-BREF: *P* < .5, single-item QoL: *P* < .5, EQ5D: *P* > .5), however, on anxiety measures, single-item QoL (*P* = .024) and WHOQOL-BREF (*P* = .006) were significantly different.

**Table 4.** Correlation Matrix for Caregiver Burden, Depression, and Anxiety Compared With Caregiver QoL Self-Reports.

QOL Measures	Spearman's rank correlation against HADS and ZBI					
	Burden		Depression		Anxiety	
	Correlation coefficient	Significance (*P*)	Correlation coefficient	Significance (*P*)	Correlation coefficient	Significance (*P*)
WHOQOL-BREF	−0.619	.003	−0.680	.000	−0.706	.000
Single-item QoL	−0.584	.007	−0.649	.001	−0.600	.002
EQ5D	−0.145	.530	−0.216	.312	−0.241	.258

Abbreviations: HADS, Hospital Anxiety and Depression Scale; QoL, quality of life; WHOQOL-BREF, World Health Organization Quality of Life–short version; ZBI, Zarit Burden Interview.

Post hoc analyses on anxiety measures showed that QoL scores (single-item QoL and WHOQOL-BREF) of those with normal anxiety (means: 4.9 and 104.3) had significantly higher mean QoL scores compared with those with borderline case (4.1 and 90.1; *Z* = −1.96, *P* = .050) and case (3.8 and 80.8; *Z* = −2.22, *P* < .05). Hierarchical multiple regression models using WHOQOL-BREF and single-item QoL as dependent variables, and depression and anxiety as independent predictors were performed. Dementia severity (CDR) was non-significant; however, depression was an independent predictor of QoL (WHOQOL-BREF: *F*_1,19_ = 20.67, *P* = .000; single-item QoL: *F*_1,18_ = 12.08, *P* = .003), accounting for 49.6% and 35.7% of variance, respectively. Anxiety accounted for 51.9% and 39.1% of QoL variance, respectively (WHOQOL-BREF: *F*_1,19_ = 22.78, *P* = .000; single-item QoL: *F*_1,18_ = 14.13, *P* = .001).

## Discussion

The present study investigated QoL in young-onset patients and caregivers using different conceptual measures of QoL and compared both self- and proxy reports. Patient QoL self-reports for the QoL-AD were higher than caregiver proxy reports, consistent with previous research by Bakker et al and Kimura et al.^25,26^ Furthermore, patient QoL self-reports for the broad (WHOQOL-BREF) and single-item measures of QoL were higher than those of caregivers. However, physical health reports (WHOQOL-BREF and EQ5D) did not differ. Caregiver burden, depression, and anxiety negatively influenced caregiver QoL when measured using the WHOQOL-BREF and single-item metric, but not when measured by the health-related instrument (EQ5D).

Why then is there a disparity between patient and caregiver QoL scores? Perhaps the most obvious and readily accepted explanation is impaired patient cognition or insight. In this view, patient QoL may in fact be worse than self-report suggests, but cognitive impairment may limit accurate perception and reporting. Although possibly observed in some LoD cohorts, justified by inconsistent longitudinal patient self-reporting,^51^ this finding is disputed.^52,53^ Regardless, YoD patients may differ from their LoD counterparts. Alternatively, patients may be accurate in reporting their QoL, yet it may be difficult for caregivers to appreciate this externally, and the caregiver’s QoL may also be affecting this perception.

The biggest disparity between patient and caregiver QoL scores was on the domain of social functioning on the WHOQOL-BREF, suggesting an impact of loss of social interaction in caregivers with consequent reduction in QoL. This finding is supported in the LoD literature^53^ and further suggested by the effect of perceived caregiver burden on QoL reports in this cohort. Alternatively, perhaps patients normalize their experience and are able to cope with all sorts of disability, coping far better (and with higher QoL) than they ever would have imagined.^54^ Future research could potentially further delineate this.

Caregiver burden, depression, and anxiety were demonstrated to have a negative influence on caregiver QoL. This knowledge suggests that maintenance and promotion of QoL should target these aspects for intervention. Tackling such factors effectively appears to be quite complex. Research by Mioshi et al^55^ of an intervention promoting coping skills in FTD caregivers suggested that caregiver anxiety may be quite resistant to modification or may require a different approach to interventions that reduce caregiver burden.

The QoL instruments used in the study were acceptable to participants, and they appeared able to understand the items. We would, however, argue that each of the QoL instruments used here lacks specificity in capturing the YoD experience, particularly in comparison with items used in other scales measuring subjective experience in YoD.^16^ For example, the scales do not measure features that we believe to be fundamental to YoD QoL such as the management of difficult symptoms; health and social care needs; loneliness/isolation; stigma; worries about finances, employment, and parenting; and decreasing social network. As such, we propose that future research try to develop a QoL measure more sensitive and specific to the context of YoD.

While this was a relatively small sample, it did have the benefit of a high level of homogeneity at the time of recruitment, with age less than 65 years at time of onset and diagnosis, the dyadic nature of the data set, and the fact that individuals were community dwelling and not in formal care. This helps with interpretation of QoL in this population and with efforts to support individuals and their carers. Future research could build on this study by further investigating dementia subtype-specific differences in QoL, developing the suggestion from Hvidsten et al^23^ that there is little difference in QoL between AD and FTD YoD groups. Further understanding of the stresses of caregivers would be useful, particularly in relation to health economic factors and involvement of social networks.

## Conclusion

The study has investigated QoL in YoD patients and caregivers. Patients tend to self-report their QoL as higher than caregiver perceptions of patient-QoL, and caregiver self-reported QoL in most areas apart from those measuring physical health status. In providing care and support for caregivers, factors such as burden, depression, and anxiety should be targets for interventions. Further research should strengthen these results by observing longitudinal trends and measuring QoL across YoD patient subsets.
